# Threonine 286 of fatty acid desaturase 7 is essential for ω-3 fatty acid desaturation in the green microalga *Chlamydomonas reinhardtii*

**DOI:** 10.3389/fmicb.2015.00066

**Published:** 2015-02-05

**Authors:** Jong-Min Lim, Jayaraman Vikramathithan, Kwon Hwangbo, Joon-Woo Ahn, Youn-Il Park, Dong-Woog Choi, Won-Joong Jeong

**Affiliations:** ^1^Sustainable Bioresource Center, Korea Institute of Bioscience and BiotechnologyDaejeon, South Korea; ^2^Department of Biological Science, Chungnam National UniversityDaejeon, South Korea; ^3^Advanced Radiation Technology Institute – Korea Atomic Energy Research InstituteJeonbuk, South Korea; ^4^Department of Biology Education, Chonnam National UniversitySouth Korea

**Keywords:** CrFAD7, Thr286, fatty acid desaturase, topology, *Chlamydomonas reinhardtii*

## Abstract

Omega-3 fatty acid desaturases catalyze the conversion of dienoic fatty acids (C18:2 and C16:2) into trienoic fatty acids (C18:3 and C16:3), accounting for more than 50% of the total fatty acids in higher plants and the green microalga *Chlamydomonas reinhardtii*. Here, we describe a Thr residue located in the fourth transmembrane domain of fatty acid desaturase 7 (FAD7) that is essential for the biosynthesis of ω-3 fatty acids in *C. reinhardtii*. The ω-3 fatty acid deficiency in strain CC-620, which contains a putative missense mutation at Thr286 of CrFAD7, was recovered by the overexpression of CC-125 *CrFAD7*. A Ser substitution in position 286 was able to partially complement the phenotype of the ω-3 fatty acid deficiency, but other substitution variants, such as Tyr, His, Cys, and Gly, failed to do so. Prediction of the phosphorylation target site revealed that Thr286 may be phosphorylated. Analysis of the structural conformation of CC-620 CrFAD7 via topology prediction (and bends in the helix) shows that this missense mutation may collapse the catalytic structure of CrFAD7. Taken together, this study suggests that Thr286 is essential for the maintaining the catalytic structure of CrFAD7.

## INTRODUCTION

Fatty acids are the primary components of cell membranes. Long-chain unsaturated fatty acids are important constituents of cellular membranes and play significant roles in maintaining membrane fluidity in many organisms. Polyunsaturated fatty acids, including α-linolenic acid, are important for low-temperature responses (Gibson et al., 1994), sperm and pollen viability (Horiguchi et al., 1996; McConn and Browse, 1996; Wathes et al., 2007), and defense signaling (Kirsch et al., 1997; Wallis and Browse, 2002) in various organisms.

Membrane glycerolipid molecules comprise high levels of trienoic fatty acids, and more than 2/3 of the fatty acids available in thylakoid membranes are C18:3 or a combination of C18:3 and C16:3 (Routaboul et al., 2012). The ω-3 fatty acid desaturases (FADs) catalyze the conversion of dienoic fatty acids (C18:2 and C16:2) into trienoic fatty acids (C18:3 and C16:3). In higher plants, three ω-3 FADs—FAD3, FAD7, and FAD8—were identified and characterized (Wallis and Browse, 2002). FAD3 is specific for the endoplasmic reticulum, whereas FAD7 and its cold-inducible isozyme, FAD8, are plastid specific (Wallis and Browse, 2002). Although a large number of different FADs have been characterized from a variety of organisms, some of the more specialized enzymes are still being discovered. In the model microalga *Chlamydomonas reinhardtii*, 60% of total fatty acids are composed of ω-3 and ω-6 polyunsaturated fatty acids, of which more than 80% are ω-3 species (Giroud and Eichenberger, 1988; Siaut et al., 2011). The CrFAD7 is the only ω-3 FAD in *C. reinhardtii* and may play a similar role in low-temperature responses as in higher plants (Nguyen et al., 2013). Recently, it was reported that the CC-620 strain is a ω-3 fatty acid-deficient mutant (Pflaster et al., 2014); the segregation analysis of CC-620 × CC-125 implicated that the deficiency is attributable to a missense mutation at Thr286 in CrFAD7.

Fatty acid desaturases are grouped into soluble and integral membrane classes in higher plants (Shanklin and Somerville, 1991). The soluble acyl-carrier-protein (ACP) desaturase enzymes are found in the plastids of higher plants, whereas the more extensive group of integral membrane acyl-CoA desaturases exist in endomembrane systems in both prokaryotes and eukaryotes (Shanklin and Cahoon, 1998). Most of the membrane-bound desaturases are homodimeric proteins that contain four membrane-spanning domains and three His box motifs [HX3-4H, HX2-3HH, and (H/Q)X2-3HH] (Shanklin, 1994). The di-iron active site of these enzymes is buried within a core four-helix bundle and positioned alongside a deep, bent, narrow hydrophobic cavity in which the substrate is bound during catalysis (Shanklin, 1994). Similarly, membrane receptors and channel proteins are integral membrane proteins composed of several transmembrane (TM) α-helices that assemble through tertiary or quaternary structures to form bundles that cross the lipid bilayer (Palczewski et al., 2000; Jiang et al., 2003). The biological function of these proteins involves conformational rearrangement of this TM bundle.

In higher plants and green algae, FAD7 also has four TM domains and His motifs. In addition, FAD7 has well-conserved Thr residues that are located distal to the active catalytic site; the roles of these Thr residues have not been studied (Shanklin, 1994; Pflaster et al., 2014). In the present study, we show that Thr286 is essential for the desaturation activity of CrFAD7 using complementation analyses with various modified FAD7s. We then discussed for the critical role of Thr286 in the maintenance of the active catalytic strucuure of CrFAD7 using prediction analysis of phosphorylation and topology. This study will help identify the physiological roles of ω-3 fatty acids in microalgae and lead to an understanding of the structure and function of membrane-bound desaturases.

## MATERIALS AND METHODS

### *C. reinhardtii* GROWTH CURVES AND CULTURE CONDITIONS

*Chlamydomonas reinhardtii* cultures were maintained on Tris-acetate-phosphate (TAP) agar medium at 25^∘^C under continuous light. A single colony of *C*. *reinhardtii* was used to make a starter culture grown in liquid TAP medium. The starter culture was grown under 4 days of light on a rotary shaker (200 rpm). Fresh TAP liquid medium (50 ml) was prepared in 250 ml Erlenmeyer flasks. This starter culture was inoculated at an OD_750_ of 0.05. The growing cells were measured daily with an UV-visible spectrophotometer at 750 nm.

### VECTOR CONSTRUCTION AND TRANSFORMATION

The coding region of *CrFAD7* (XM_001689611) cDNA was amplified by reverse transcription PCR (RT-PCR) using the primers 5^′^-CAT ATG CAG TGC CTG TCT CGC TCC A-3^′^ and 5^′^-GGGATATCTTAGGCCTTGCCGGCAA-3^′^ (restriction sites underlined). CrFAD7 genomic DNA was amplified by PCR using the following primers: 5^′^-ACAT ATG CAG TGC CTG TCT CGC TCC A-3^′^ and 5^′^-AGAT ATC GCC GTG CCA GAG TCT AAC T-3^′^ (restriction sites underlined). The *CrFAD7* cDNA and genomic DNA were cloned into expression vector pCr112 at the *Nde*I and *Eco*RV sites. This construct consists of the *psa*D promoter, *CrFAD7*, and the *psa*D terminator along with the hygromycin resistance gene for selection, which is regulated by the *β-tub* promoter and the *rbcS2* terminator. The construct was transformed into strain CC-620 using the glass bead method (Kindle, 1990). After transformation, cells were cultured in the dark without shaking for 2 days. The selection procedure was performed on agar medium containing 15 μg/ml hygromycin, and the plates were incubated under dim light for2 weeks.

### RNA AND DNA ISOLATION

Total RNA was prepared from *C. reinhardtii* cells (∼ 1 × 10^7^ cells) using the TRIzol reagent (Invitrogen, Carlsbad, CA, USA) according to the manufacturer’s instructions. Five micrograms of total RNA were treated with 1 U RNase-free DNase (TaKaRa, Osaka, Japan) for 30 min, and purification was performed according to the manufacturer’s instructions. For the isolation of genomic DNA, 500 μl TEN buffer (10 mM EDTA, 10 mM Tris-HCl, and 150 mM NaCl) was added to samples (∼ 1 × 10^7^ cells). Cells were harvested and resuspended in 150 μl H_2_O and 300 μl SDS-EB buffer (2% SDS, 400 mM NaCl, 100 mM Tris-HCl, pH 8.0, and 40 mM EDTA). Extraction was carried out first with 350 μl phenol:chloroform:isopropanol (24:24:1, v/v/v) and then with 300 μl chloroform:isopropanol (24:1, v/v). Genomic DNA was precipitated with two volumes of absolute ethanol and washed with 70% ethanol. The DNA pellet was resuspended in Tris-EDTA buffer.

### RT-PCR ANALYSES

Reverse transcription was carried out using 2 μg total RNA, 50 μM oligo (dT), 200 U M-MLV reverse transcriptase (Promega, Madison, WI, USA), 500 μM each dNTP, and 20 U ribonuclease inhibitor. For semi-quantitative RT-PCR, cDNA was amplified with 1.5 U Ex *Taq* DNA polymerase (TaKa Ra), 100 μM each dNTP, and 10 pmol each gene-specific primer using a T1 thermal cycler (Biometra GmbH, Gottingen, Germany). Amplification was performed with 25–35 cycles of 94^∘^C for 30 s, 56^∘^C for 30 s, and 72^∘^C for 30 s. To detect transcripts, the following primers were used: *CrFAD7* forward primer: 5^′^-TTCACCGCTGAGCGCAA-3^′^; reverse primer: 5^′^-GTGGGTGCCGATGTCGTGGT-3^′^; *CrFAD2* forward primer: 5^′^-CTTCACCAAGCGCGAGCGCA-3^′^; reverse primer: 5^′^-CTTGATGGCCTCGGTGGCCT-3^′^; *CrΔ4FAD* forward primer: 5^′^-CACCTTCGCCGTGTCGCACA-3^′^; reverse primer: 5^′^-CATCTCGCCGTCGCGCTTGA-3^′^; *CrFAD6* forward primer: 5^′^-ATGGCCAAGTGGGACTCCAC-3^′^; reverse primer: 5^′^-CCACGGTGAAGGTGCTCATC-3^′^; *CrFAD13* forward primer: 5^′^-CATGATCTCGCCCCTTAGCTACTT-3^′^; reverse primer: 5^′^-GTCCATCTGAATGTGGGACACCT-3^′^; *α-tubulin* forward primer: 5^′^-CTCGCTTCGCTTTGACGGTG–3^′^; reverse primer: 5^′^-AACGTCCTTGGGCACGACGT -3^′^; and *IAD5* forward primer: 5^′^-GCGAGGTCTCTGCTCTGGTG-3^′^; reverse primer: 5^′^-TACTCGGACTTGGCGATCCA -3^′^. To detect *CrFAD7* cDNA transcripts in transgenic lines, specific primers [forward 5^′^-TTCACCGCTGAGCGCAA-3^′^ located in *CrFAD7* cDNA and reverse 5^′^-CCTGTGGCTAATTGACCGTG-3^′^ located in psaD terminator] were used.

### ANALYSIS OF FATTY ACID METHYL ESTERS BY GAS CHROMATOGRAPHY

Total lipids were extracted from 20 mg freeze-dried samples according to a previously described extraction method (Sasser, 1990). Saponification was performed with 2 ml saponification reagent (7.5 M NaOH:CH_3_OH, 1:1, v/v) at 100^∘^C for 30 min. For the production of fatty acid methyl esters, 4 ml methylation reagent (CH_3_OH:6 N HCl, 1:1, v/v) were added to the saponified sample and incubated at 80^∘^C for 10 min. After the reaction, 2.5 ml extraction solvent hexane:methyl tetra-butyl ether, 1:1, v/v) were added and incubated with shaking for 10 min. The upper phase was separated by centrifugation at 4,000 rpm for 10 min. A washing step was carried out with 6 ml washing solution (0.5 M NaOH). The fatty acid methyl esters were analyzed by gas chromatography (model YL-6100GC; Young Lin Science, Anyang, Korea) equipped with a flame ionization detector and an INNOWAX capillary column (Agilent Technologies, Santa Clara, CA, USA; 30 m × 0.32 mm × 0.5 μm). Each fatty acid methyl ester component was identified and quantified using the Supelco®; 37 Component Fatty Acid Methyl Ester Mix (Sigma).

### SOUTHERN AND NORTHERN ANALYSES

Ten micrograms of genomic DNA were digested with *Kpn*I and separated by 0.8% agarose gel electrophoresis. The separated DNA was blotted onto a Hybond N^+^ nylon membrane (Amersham Biosciences, Piscataway, NJ, USA). A 0.3-kb PCR fragment corresponding to the *C*-terminal region of *CrFAD7* was used as a probe. ^32^P-labeled probes were produced using the Rediprime^TM^ II Random Labeling System (Amersham Biosciences), and hybridization was performed according to the manufacturer’s instructions. Signals were detected using the Bio-Imaging Analyzer BAS-1800II (Fuji, Tokyo, Japan).

Total RNA was extracted from *C. reinhardtii* cells (5 ml liquid culture) using the TRIzol reagent (Invitrogen, Carlsbad, CA, USA) according to the manufacturer’s instructions. Twenty micrograms of total RNA were separated by gel electrophoresis and blotted onto a Hybond-N nylon membrane (Amersham Biosciences, USA) by capillary transfer. A 0.3-kb fragment corresponding to the CrFAD7 cDNA was used as a probe. Procedures for hybridization and signal detection were as described above.

#### *IN SILICO* ANALYSES

To identity conserved Thr residues of *C. reinhardtii* FAD7, we used CrFAD7 as bait and employed the BLASTP program (BLASTP 2.2.26+) hosted at Phytozome, version 9.1 (). Clustal W2.1 was used for multiple sequence alignment. The accession numbers of the FAD7-homologous genes used in this study were as follows: *VcFAD7* (XP_002953984), *CnFAD7* (EF1N50714), *CosFAD7* (EIE21058), *CrFAD7* (XP_001689663), *OsFAD7* (NP_001060733), *PpFAD7* (XP_001752878), *AtFAD7* (NP-187727), *GmFAD7* (NP-001237838), *ZmFAD7* (NP-001105303), and *SspDesA* (CAA37584). Prediction analyses for phosphorylation target sites and topology were performed using the online software tools Kinase phos2 and TMHMM (Version 1), respectively. Default settings of both programs were used.

## RESULTS

### THE ω-3 FATTY ACID DEFICIENCY IN THE CC-620 STRAIN IS COMPLEMENTED BY CC-125 *CrFAD7* GENE EXPRESSION

**FIGURE 1:**
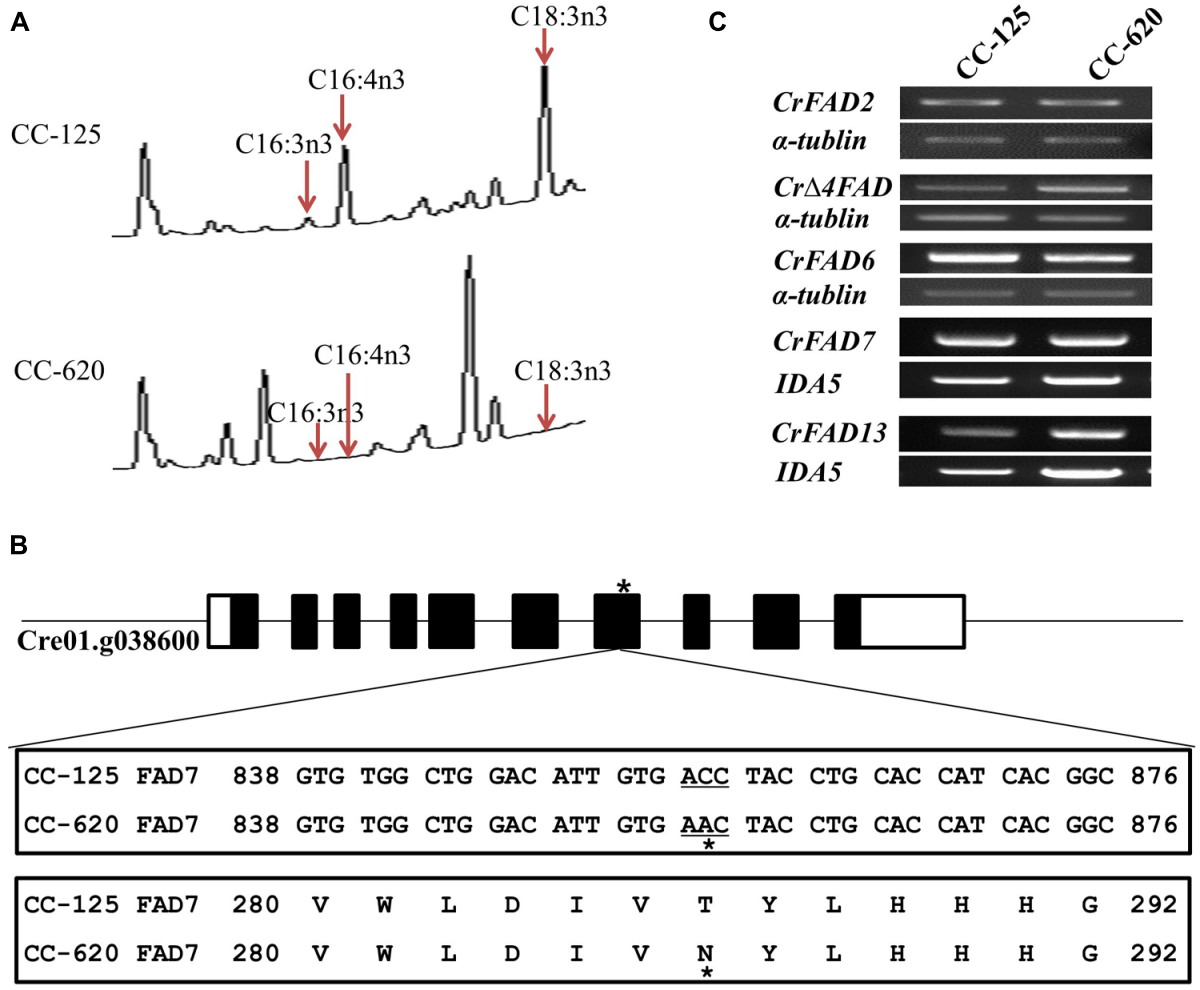
**(A)** Comparison of fatty acids profiles in strains CC-125 and CC-620 from gas chromatographic analyses. Arrows indicate significant changes in specific fatty acids. **(B)** Sequence comparison (nucleotide and amino acid) of CrFAD7 in CC-620 and other wild-type strains (CC-124, CC-125, and CC-621). The black square boxes indicated the exon number or gene locus of the CrFAD7 and star indicates the missense mutation (Thr286Asn) caused by a point mutation. **(C)** RT-PCR analyses of fatty acid desaturases (FADs). *CrFAD2* (EU596472), *CrFAD7* (XM-001689611), *CrFAD6* (AB007640), *CrFAD13* (AB239770), and *Δ-4FAD* (JN089704). Genes for *IAD5* and *α-tubulin* served as internal controls.

We confirmed the ω-3 fatty acid deficiency in the CC-620 strain obtained from the Chlamydomonas Resource Center (**Figure 1A**). The point mutation in the seventh exon of the *CrFAD7* gene, which causes a missense mutation (Thr286Asn), was confirmed to be identical to that described in the study by (Pflaster et al., 2014; **Figure 1B**). To identify the main cause for abnormal ω-3 fatty acid desaturation, transcript levels were investigated for *CrFAD2* (EU596472), *CrΔFAD4* (JN089704), *CrFAD6* (AB007640), *CrFAD7* (XM_-_001689611), and *CrFAD13* (AB239770), whose expression products are either directly or indirectly involved in ω-3 fatty acid biosynthesis. However, RT-PCR analysis detected no significant differences in the expression levels of these genes between the CC-620 and CC-125 strains (**Figure 1C**). Next, we sequenced the cDNAs of the above-mentioned genes. Except for the point mutation in the *CrFAD7* gene, no mutations were observed in the *CrFAD2*, *CrΔFAD4*, *CrFAD6*, or *CrFAD13* genes.

**FIGURE 2:**
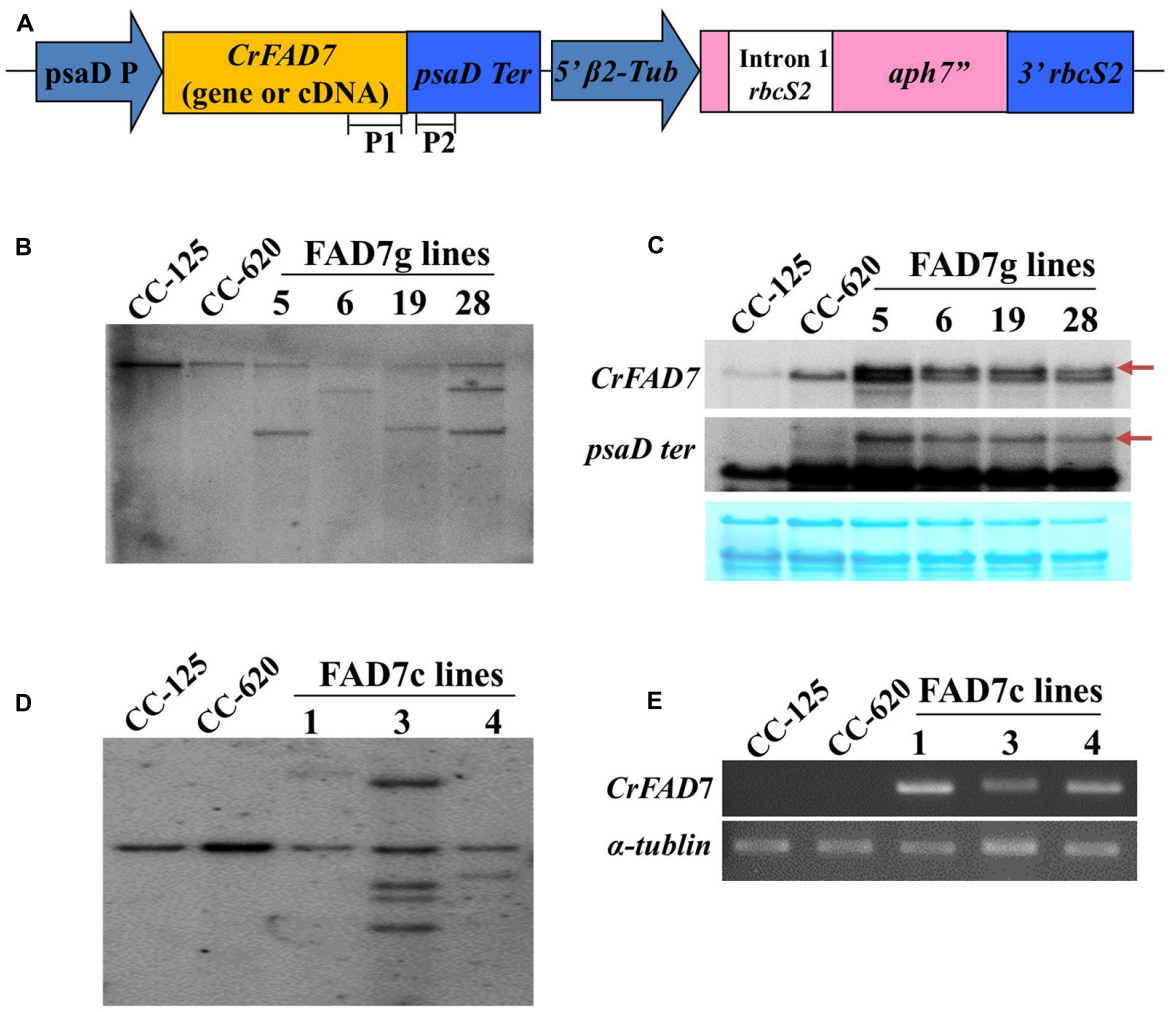
**Generation of CC-125 *CrFAD7*-expressing transgenic lines using CC-620. (A)** Schematic diagram of the transformation vector. P1 and P2 indicate the regions used as probes for Southern or northern blot analysis. **(B,D)** Southern blot analysis of the *CrFAD7* gene. Genomic DNA was digested by *Xba*I and blotted against probe P1. **(C)** Northern blot analysis to identify the expression of *CrFAD7* mRNA in the *CrFAD7* genomic DNA-expressing transgenic lines; P1 probe for *CrFAD7* transcript or P2 probe for 3^′^ UTR (*PsaD* terminator) of *CrFAD7* transcript. Arrows indicate transcripts of transformed *CrFAD7* gene. **(E)** RT-PCR analysis to identify transcripts of transformed *CrFAD7* cDNA. *α-tubulin* served as the internal control. FAD7g lines: complementation lines with FAD7genomic DNA; FAD7c lines: complementation lines with FAD7 cDNA.

To reveal the phenotype of the ω-3 fatty acid deficiency in strain CC-620 by the missense mutation detected in *CrFAD7*, genetic transformation of CC-620 was carried out using genomic DNA and cDNA of CC-125 *CrFAD7*. Transgene insertion and expression were confirmed by Southern, Northern, and RT-PCR analyses (**Figures 2A–E**).

**FIGURE 3:**
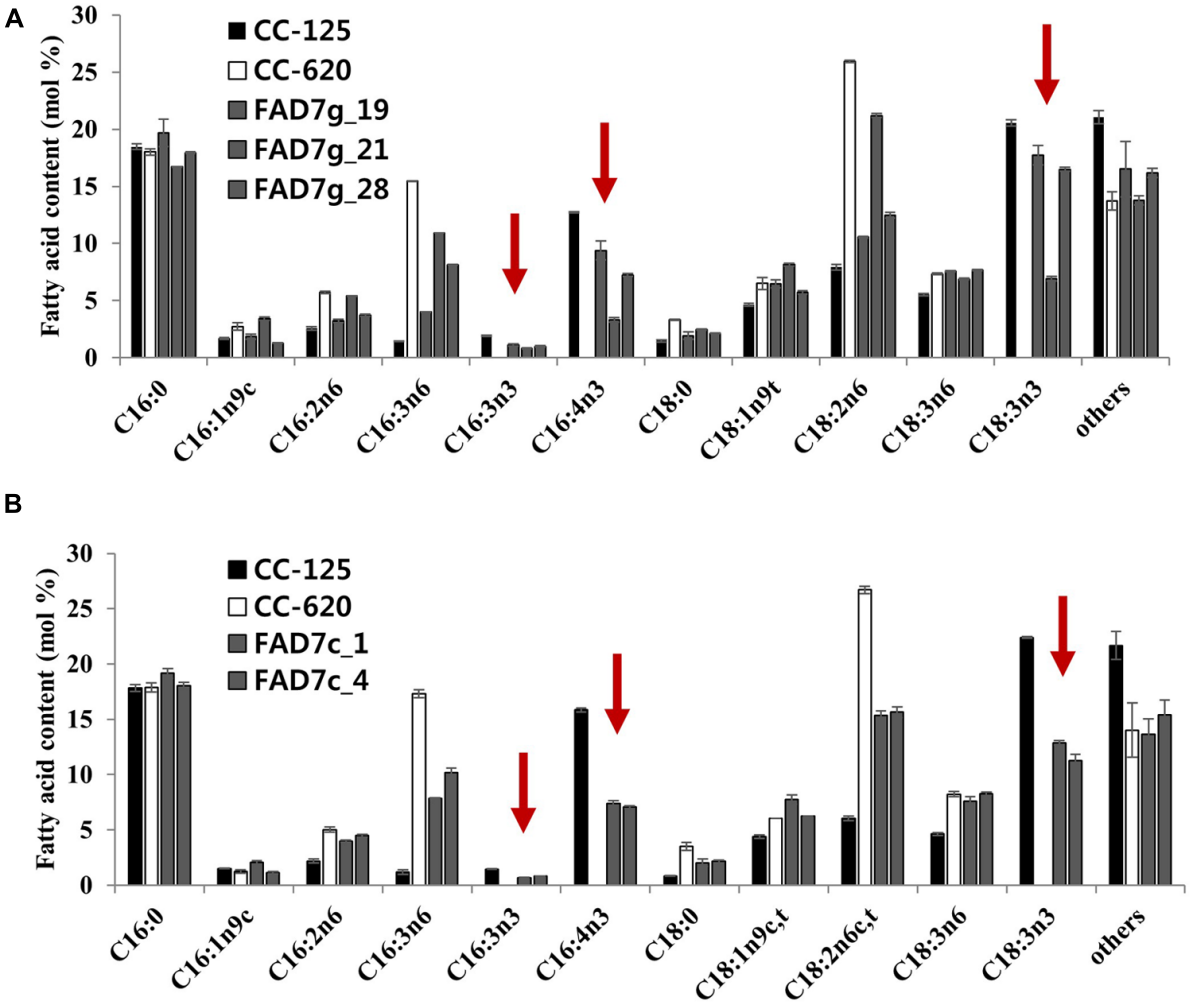
**Complementation of ω3-fatty acid deficiency in strain CC-620 by expression of the CC-125 *CrFAD7* gene.** Fatty acid compositions in CC-125, CC-620, and complementary strains were compared. **(A)** Complementation using *CrFAD7* genomic DNA. FADg_19, FADg_21, and FADg_28 complementation strains expressing *CrFAD7* genomic DNA. **(B)** Complementation using *CrFAD7* cDNA. FAD7c_1 and FADc_4 complementation strains expressing *CrFAD7* cDNA. The values represent the mean ± standard error (SEM). The experiments were conducted in triplicate.

In *CrFAD7* genomic DNA-complemented strains (in transgenic lines), the normal fatty acid composition was recovered with various levels of C16:4 and C18:3n3; in comparison with CC-125, more than 80% of ω-3 fatty acids were recovered with a concomitant reduction of ω-6 fatty acids in several transgenic lines (**Figures 3A,B**). The ω-3 fatty acids hexadecatrienoic acid (C16:3n3), hexadecatetraenoic acid (C16:4), and α-linolenic acid (C18:3n3) were not detected in the CC-620 strain, whereas, the ω-3 fatty acids were found to be 1.9, 12.7, and 20.5 mol% in CC-125, and to be 1.1, 9.3, and 17.7 mol% in the complemented strain (**Figure 3**; **Table 1**). The concomitant reduction in ω-6 fatty acids was detected in the complemented transgenic lines compared to the CC-620 strain, but no significant differences in either saturated (C16:0) or monounsaturated fatty acid accumulation were observed in CC-125, CC-620, or the transgenic lines. In *CrFAD7* cDNA-complemented strains, the recovery level of ω-3 fatty acids was lower than that of *CrFAD7* genomic DNA-complemented lines (**Figures 3A,B**).

**Table 1:** Fatty acid composition of CC-125, CC-620 and one of complemented strains analyzed through GC-FID.

Fatty acid composition	CC-125	CC-620	FAD7g_19
C16:0	18.48 ± 0.251	18.01 ± 0.292	19.68 ± 1.188
C16:1n9c	1.67 ± 0.074	2.73 ± 0.336	1.86 ± 0.22
C16:2n6	2.57 ± 0.13	5.7 ± 0.098	3.21 ± 0.148
C16:3n6	1.44 ± 0.037	15.47 ± 0.018	4 ± 0.036
C16:3n3	1.93 ± 0.058	ND	1.11 ± 0.093
C16:4n3	12.73 ± 0.067	ND	9.38 ± 0.83
C18:0	1.47 ± 0.093	3.32 ± 0.061	1.93 ± 0.327
C18:1n9t	4.63 ± 0.129	6.5 ± 0.533	6.46 ± 0.357
C18:2n6	7.93 ± 0.246	25.95 ± 0.089	10.56 ± 0.083
C18:3n6	5.52 ± 0.104	7.33 ± 0.083	7.57 ± 0.039
C18:3n3	20.56 ± 0.296	ND	17.74 ± 0.852
others	21.07 ± 0.574	13.73 ± 0.794	16.51 ± 2.434
FAME	100 ± 2.059	98.74 ± 2.555	100.01 ± 6.607

ND, not detected.

### Thr286 IS CONSERVED IN HIGHER PLANTS AND GREEN ALGAE

Fatty acid desaturase 7 is a membrane-bound desaturase, but its crystal structure has not yet been solved. Analysis of conserved sequence motifs in FAD7 shows that the four membrane-spanning domains and three His box motifs are conserved in all FAD7 from microalgae to higher plants (Figure S1). Interestingly, the Thr residue in the fourth TM domain is specifically well conserved (Figure S1). The membrane protein topology of CrFAD7 was predicted using TMHMM. Topology prediction of CrFAD7 detected clear differences between CC-125 and CC-620 CrFAD7 (Figures S2A,B). A substitution of Ser286 for Thr286 showed a similar pattern as CC-125 CrFAD7 (Figures S2A,C). In CC-125 and Ser286-substituted CrFAD7, all four TM helices were linearly arranged, and the *C*-terminal region containing the third His box motif after the fourth TM domain was predicted to be located inside the membrane (Figures S2A,C). However, in CC-620 CrFAD7, the *C*-terminal region was predicted to be located outside of the membrane (Figure S2B), indicating that the fourth TM domain changes (or loses) its structural helix.

**FIGURE 4:**
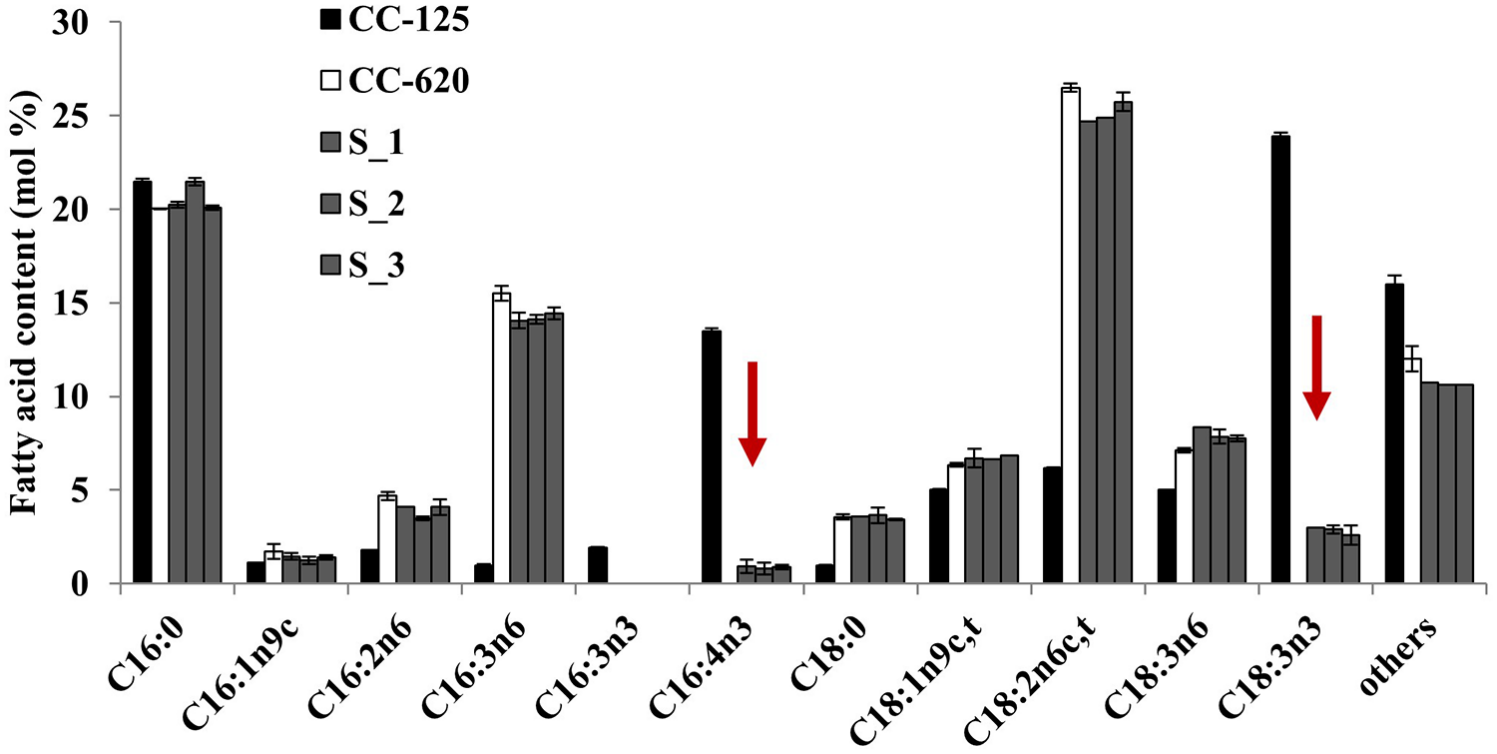
**Fatty acid composition of strain CC-620 and serine substituted lines (S_1, S_2, and S_3) expressing *CrFAD7* cDNA.** The values represent the mean ± standard error (SEM). The experiments were conducted in triplicate.

To test the above prediction that Thr could be replaced by Ser, we transformed the CC-620 strain with modified CrFAD7 genes that would encode Ser, Tyr, His, Cys, or Gly substitutions at position 286. Only the Ser-substituted transgenic lines recovered the missing fatty acids (**Figure 4**) but other substitution variants, such as Tyr, His, Cys, and Gly, failed to do so (Figure S3). However, the accumulation of fatty acids was partially complemented compared with CC-125. The accumulation of α-linolenic acid approached 3% in the Ser-substituted transgenic lines, whereas the accumulation approached 24% in strain CC-125. These results suggest that Thr286 is critical for proper CrFAD7 activity. To address the possible phosphorylation of Thr286 *in silico*, amino acid sequences of CC-620 and CC-125 CrFAD7 were analyzed using a phosphorylation site prediction tool, Kinase phos2. The result predicted 21 sites of phosphorylation, including Thr286, in CrFAD7 targeted by protein kinase B or G protein-coupled receptor kinase (Figure S4A). Only 20 target sites were predicted in CrFAD7 of strain CC-620 because of the absence of Thr286 in the CrFAD7 CC-620 strain (Figure S4B). Ser286 was predicted as a phosphorylation target for AKT1, a type of protein kinase B, but not for G protein-coupled receptor kinase (data not shown). In addition, the conserved Thr residues of all FAD7s (mentioned in Figure S1) were predicted to be phosphorylation targets (data not shown).

## DISCUSSION

In this study, our complementation results with the modified FAD7s indicated that Thr286 is essential for CrFAD7 desaturation activity (**Figures 3 and 4**). Topology and phosphorylation prediction showed that the missense mutation may disrupt the catalytic structure of the ω-3 FAD (Figures S2 and S4).

*Chlamydomonas reinhardtii* strain CC-620 is a high-efficiency mating strain that produces autolysin and has been used previously as a common laboratory wild-type strain. Recently, Pflaster et al. (2014) reported that the ω-3 fatty acid deficiency in *C. reinhartii* strain CC-620 correlated with a single locus, possibly attributed the missense mutation (Thr286Asn) in CrFAD7. In the present study, our complementation results provide direct evidence that the missense mutation is the real cause of the ω-3 fatty acid deficiency in CC-620 (**Figures 3 and 4**). ω-3 fatty acids deficiency of CC-620 can be recovered by nuclear transformation of a wild type (CC-125) *CrFAD7* gene. Interestingly, when a cDNA encoding CC-125 *CrFAD7* was used for complementation, the transformed CC-620 produced 60% ω-3 fatty acids compared to strain CC-125, and transcription could be detected by RT-PCR. However, when genomic DNA encoding CrFAD7 was used, several transformed strains produced nearly maximal levels of ω-3 fatty acids, and transcription was clearly detected by Northern blot analyses (**Figure 2C**). These results suggest that a genomic fragment corresponding to CrFAD7 efficiently expresses the CrFAD7 transcript compared to cDNA, and that the transcript level of CrFAD7 is proportional to FAD7^′^s activity.

Impaired progeny production was observed when strain CC-620 was mated with its opposite mating type (Goodenough et al., 2007). Interestingly, the involvement of ω-3 fatty acids in reproduction was also reported in higher plants and animals. The *Arabidopsis* triple mutant for FAD3, 6, and 7 manifested as male sterility (McConn and Browse, 1996). The animal ω-3 fatty acid deficiency resulted in impaired steroid hormone synthesis (Wathes et al., 2007). These findings suggest that ω-3 fatty acids may be important for reproduction in microalgae, including *C. reinhardtii*. The strain CC-620 had the additional phenotypes of slow growth and increased sensitivity at high temperature (37^∘^C) compared to the CC-125 strain (Figure S5). However, these phenotypes were recovered in the progenies of CC-620 × CC-124, which are missing ω-3 fatty acids, indicating that the CC-620 genome contains another mutation(s) responsible for these phenotypes in addition to the FAD7 mutation.

Protein phosphorylation plays a role in various physiological responses by altering enzymatic activity, from active to inactive or vice versa (Hunter, 1995). The *in silico* prediction detected 21 and 20 Thr residues of CC-125 and CC-620 CrFAD7, respectively, as target phosphorylation sites for protein kinase B or G protein-coupled receptor kinase (Figure S4). Ser286 was predicted as a phosphorylation site. However, the replacement of Thr286 by Ser286 only partially recovered the activity of FAD7 in four transgenic lines (**Figures 4** and S3). These results suggest that Thr and Ser at position 286 can be phosphorylated by kinases and that Thr is preferred and necessary for full activity of the FAD. A significant preference for Thr or Ser as the phosphoacceptor residue has been previously reported, but this selectivity and its mechanism is unknown (Marin et al., 1986; Kuenzel et al., 1987; Litchfield et al., 1990; Kõivomägi et al., 2013).

### POSSIBLE MECHANISMS OF CrFAD7 IN ω-3 FATTY ACID SYNTHESIS

In higher plants and microalgae, lipid-linked acyl desaturation is a well-known mechanism; this process is induced by FADs. All FADs have TM domains, His motif boxes, and a di-iron active site, which all play important roles in the desaturation process. Our results indicate that the conserved Thr286 in the fourth TM domain is essential for fatty acid desaturation. Thr is less polar than Ser, but both have similar structures (both contain a polar hydroxyl group), differing only in the presence of a methyl group attached to the β-carbon in Thr. A mutation of a polar amino acid in a TM helix has been reported to abolish its structure; Thr residues function cooperatively to maintain the normal structure of the TM domain through hydrogen bonding (Dawson et al., 2002). In our transgenic lines, due to the lack of this methyl group in Ser, Ser286 may not efficiently donate hydrogen molecules to maintain the correct structure of the TM domain and fails to recover the full activity of FAD7. The Thr286Asn missense mutation of the CrFAD7 protein may disrupt the structure of the TM domain, leading to a loss of CrFAD7 desaturation activity.

Mutation of Thr may directly affect the structure of the TM domain, as this single residue was able to stimulate significant helix associations in model peptides (Gratkowski et al., 2001). In the TM domain of β2-adrenergic receptors, the statistical *g*2 conformations of both Ser and Thr residues decreased their f angles and increased their c angles; a hydrogen bond was formed between the Oγ atoms of Ser and Thr, with the i-3 or i-4 carbonyl oxygen, inducing or stabilizing the angle in the helix 3–4^∘^ larger than for Ala (Ballesteros et al., 2000). This finding indicates that the local alterations in Ser and Thr in the TM domain may result in significant conformational changes across TM helices.

Based on our results, the loss of function in CC-620 CrFAD7 could be explained by a number of mechanisms. In CC-620 CrFAD7, the Thr286Asn mutation in the fourth TM domain may induce alteration of angle in the α-helix. This altered bending would induce a change in CrFAD7^′^s structural orientation. Structural shifts could deform the cooperative catalytic structure or cause the third *C*-terminal his motif box to be positioned too closed or too far from the catalytic active site. Alternately, the mutation may abolish the helix completely. Membrane topology prediction of the CC-620 CrFAD7 using TMHMM failed to detect a fourth helix. This would cause the third *C*-terminal his box to be located outside of membrane rather than inside (Figure S2B). In either scenario, the structural loss or alteration of the fourth TM domain would collapse the cooperative catalytic structure needed for ω-3 fatty acid desaturation. In addition, the presence or absence of position 286 phosphorylation could also induce a structural change in the fourth TM domain and alter the topology. CrFAD7 Thr286 seems to be positioned away from the catalytic site, indicating that the phosphorylation may cause a structural change in the fourth TM domain rather than directly regulating enzymatic activity. To support this, previous studies have shown that phosphorylation by kinase can alter the protein conformation and modulate its biological function (Birck et al., 1999; Groban et al., 2006; Grosely et al., 2013). Any of these three possible mechanisms would lead to TM domain structural modification and prevent CC-620 CrFAD7 from synthesizing ω-3 fatty acids.

In summary, we show that the *CrFAD7* point mutation directly causes an alteration in the fatty acid profile of the CC-620 strain using complementation analysis with a CC-125 copy of *CrFAD7*. Amino acid substitutions and predictions of phosphorylation sites revealed that Thr286 may be a target of phosphorylation by protein kinase B or G protein-coupled receptor kinase. Both TM topology prediction and the structure of the TM α-helix show that Thr286 may be essential for maintaining the correct catalytic structure of CrFAD7.

## Conflict of Interest Statement

The authors declare that the research was conducted in the absence of any commercial or financial relationships that could be construed as a potential conflict of interest.
