# Supplementary figures and images for: Threonine 286 of fatty acid desaturase 7 is essential for ω-3 fatty acid desaturation in the green microalga Chlamydomonas reinhardtii
Source: Front Microbiol. 2015 Feb 5;6:66. doi: 10.3389/fmicb.2015.00066 (PMC4318421; doi:10.3389/fmicb.2015.00066)

## Slide 1
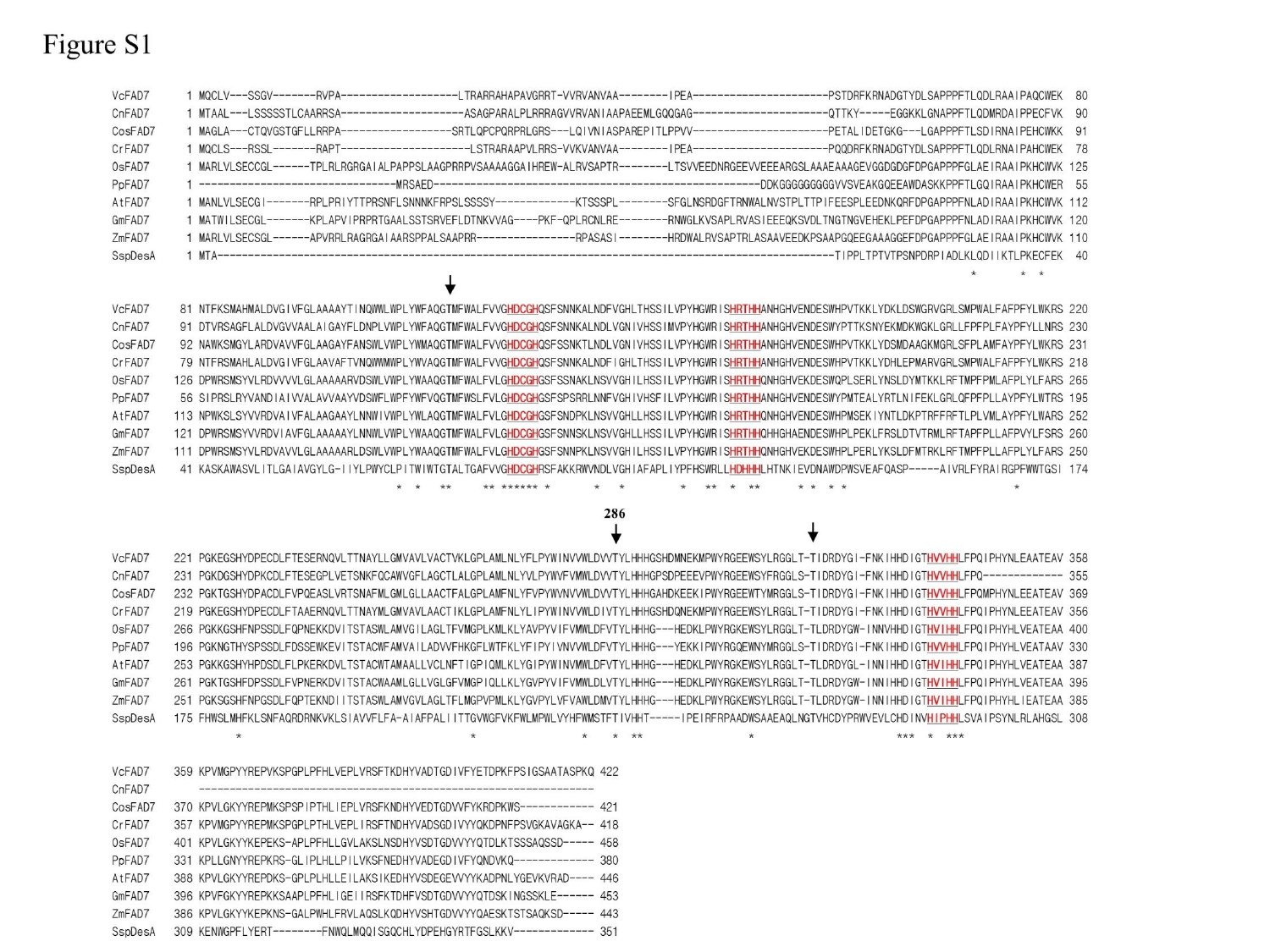

## Slide 2
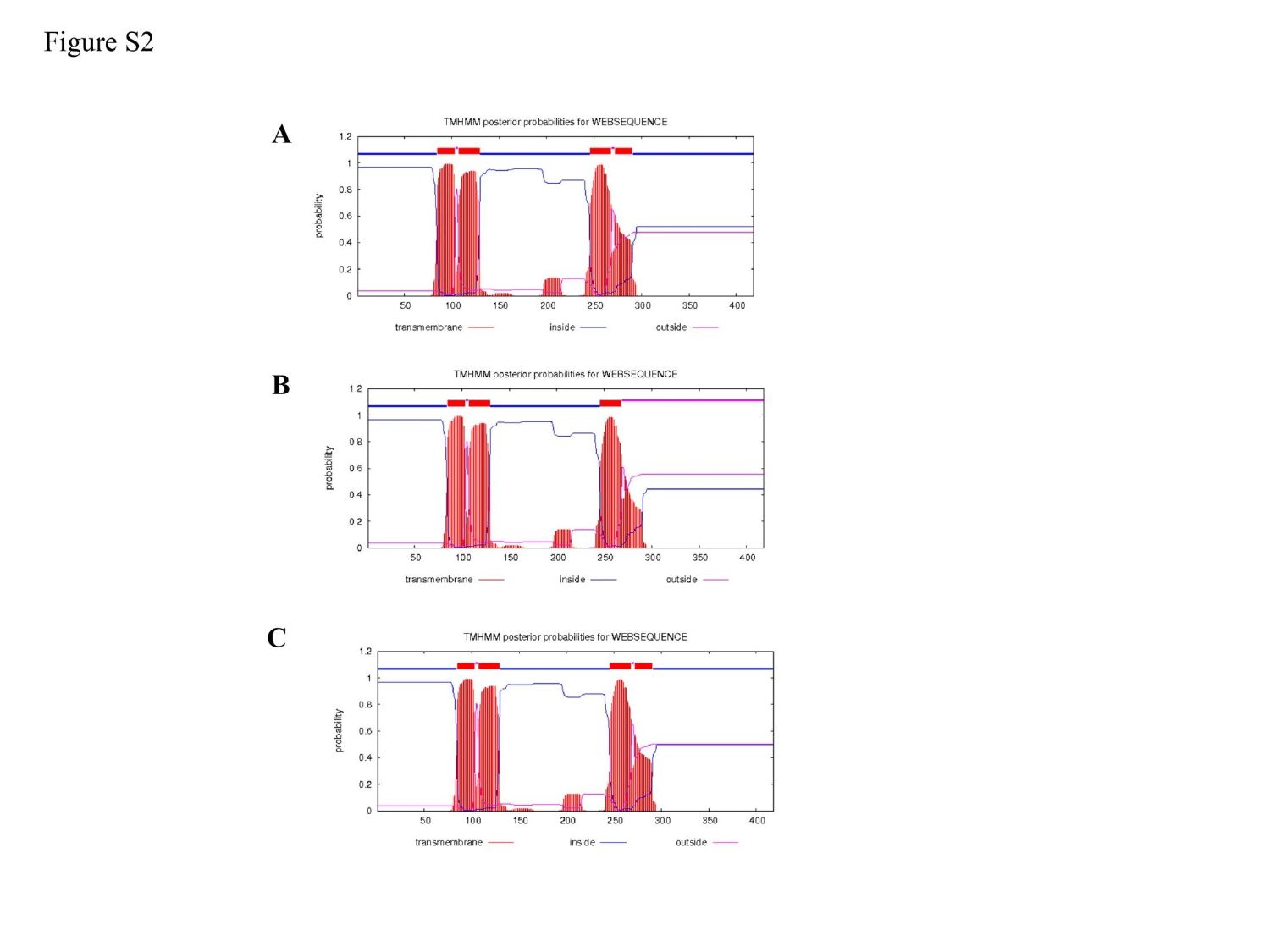

## Slide 3
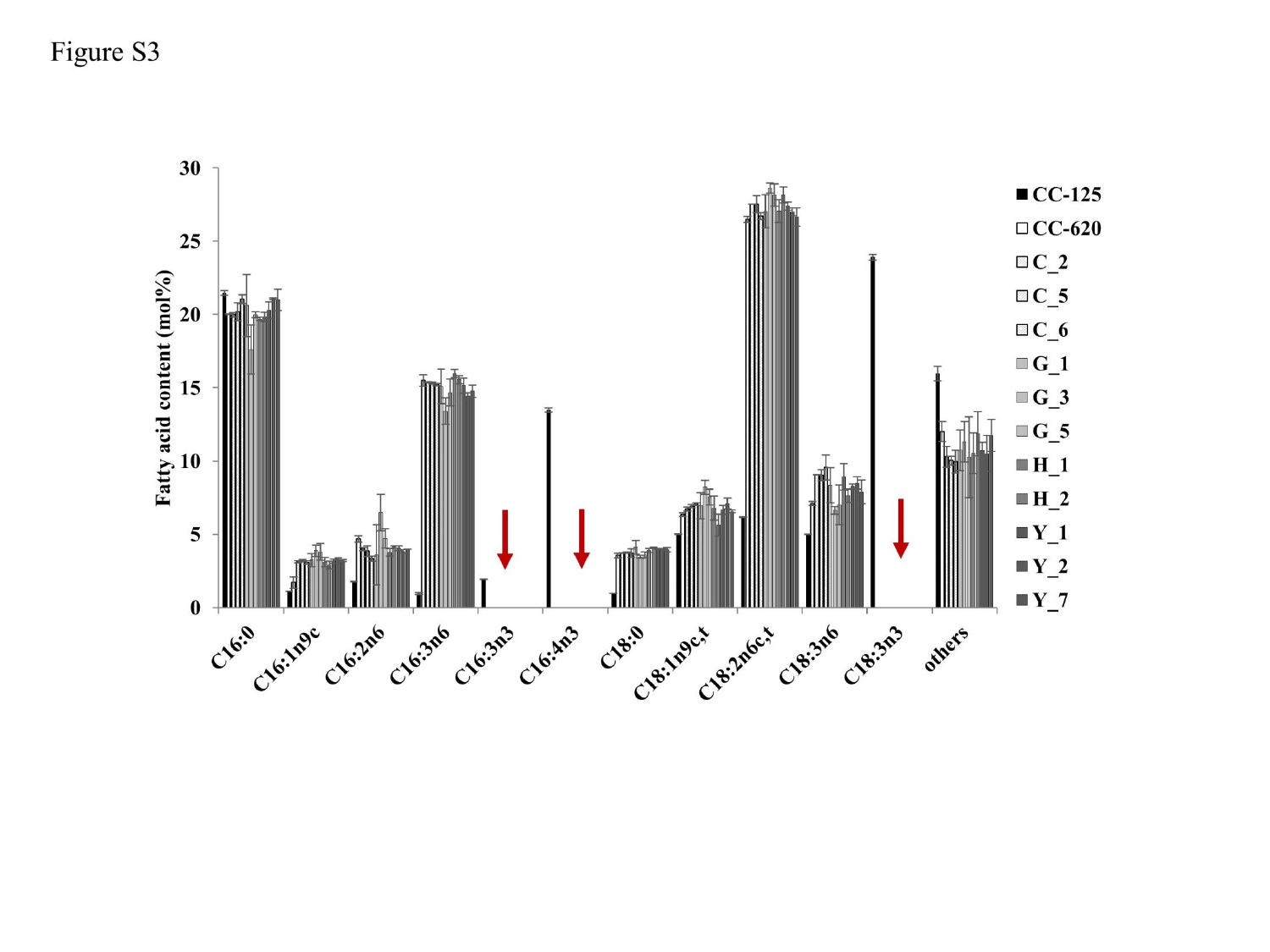

## Slide 4
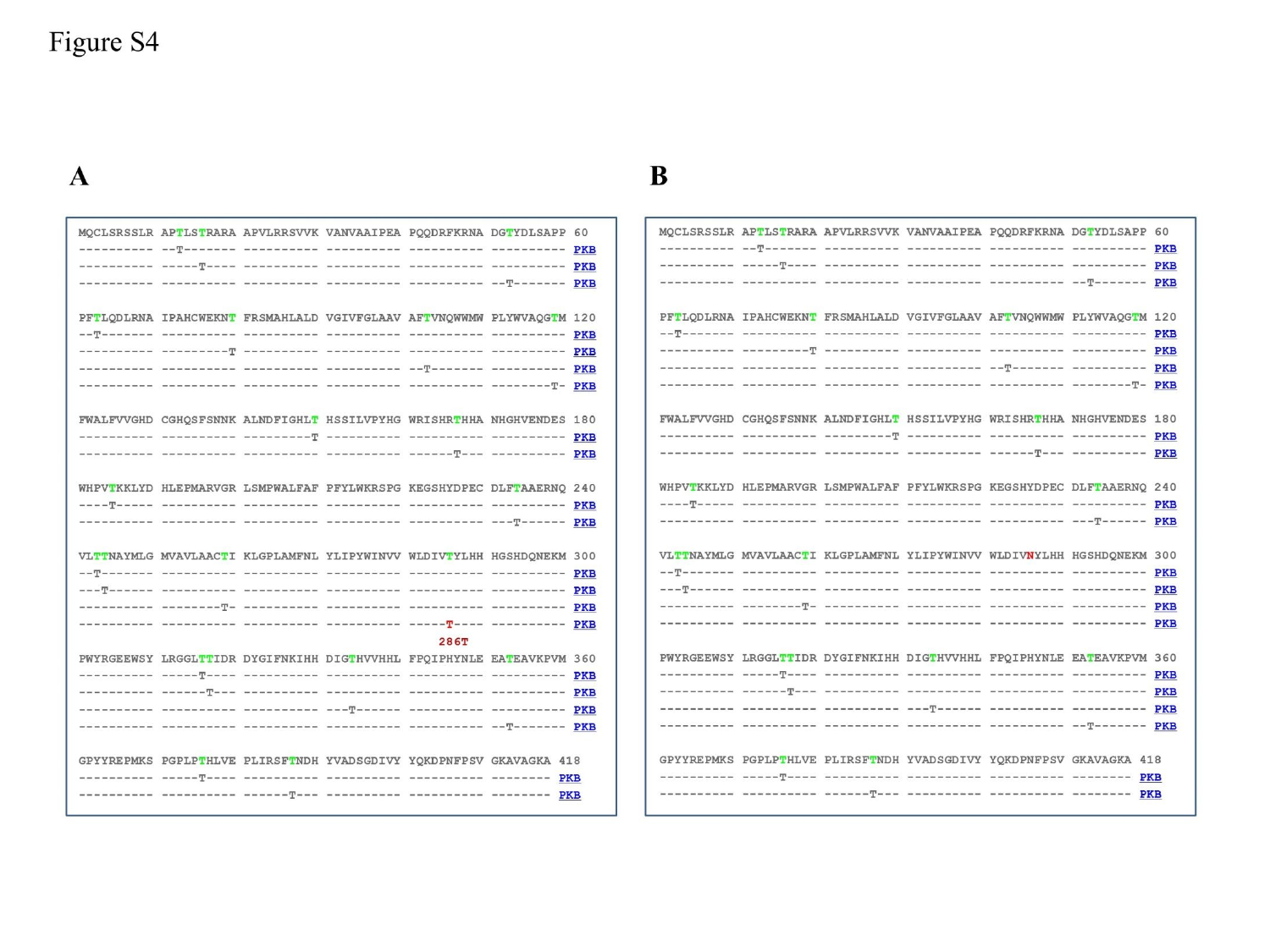

## Slide 5
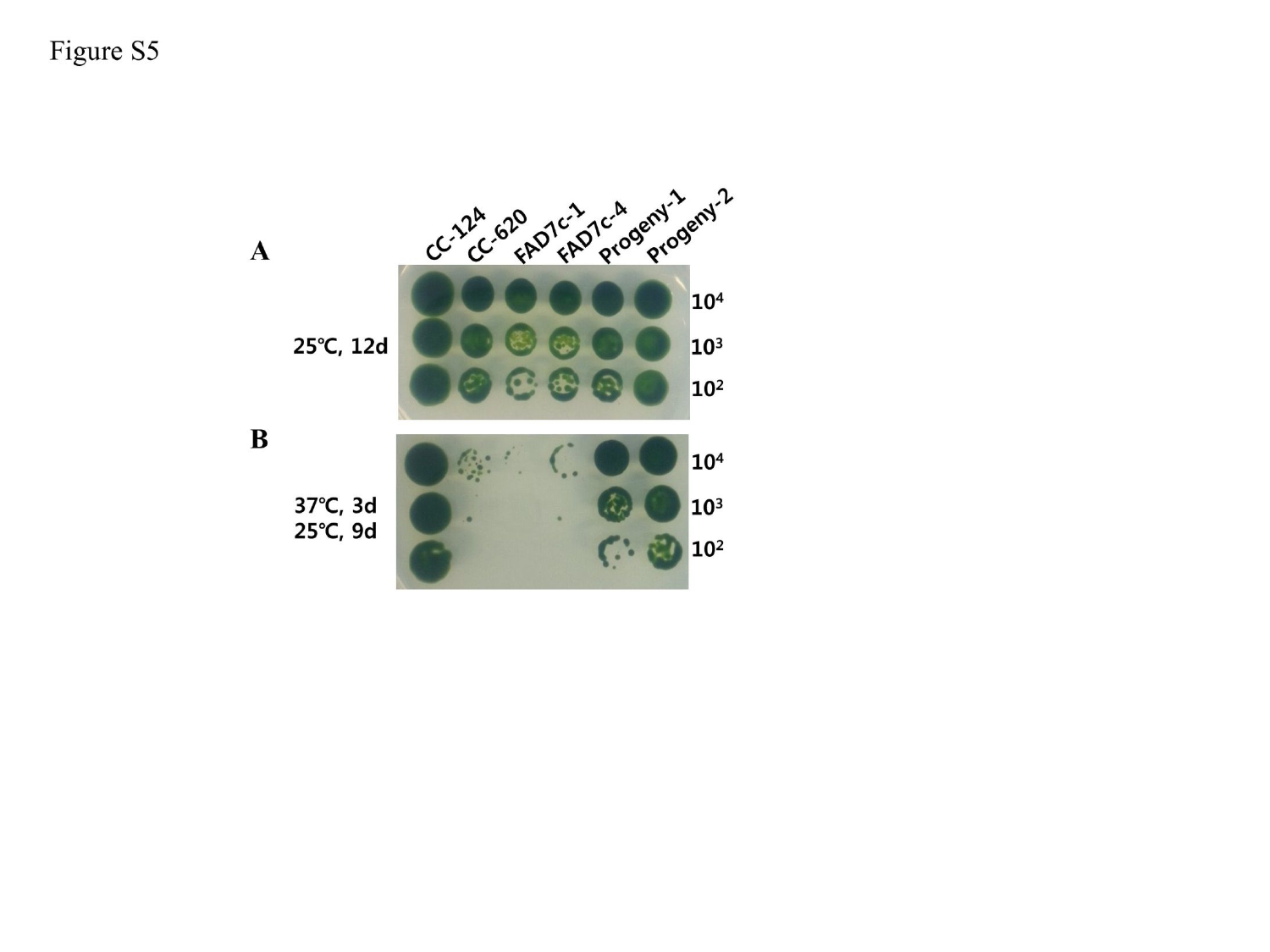

Supplement: Supplementary file 1 [file Presentation_1.PPTX]
